# GWAS combined with QTL mapping reveals the genetic loci of leaf morphological characters in *Nicotiana tabacum*

**DOI:** 10.1186/s12870-024-05261-8

**Published:** 2024-06-20

**Authors:** Yan Ji, Guoxiang Liu, Sifan Yan, Xun Jiang, Mengting Wu, Wei Liu, Yuan Li, Aiguo Yang, Peigang Dai, Shuaibin Du, Yangyang Li, Jun Wang, Xingwei Zhang

**Affiliations:** 1 Tobacco Research Institute, Chinese Academy of Agricultural Sciences, Qingdao, CN-266000 China; 2 Ruijin Branch, Jiangxi Ganzhou Tobacco Company of China Tobacco Corporation, Ganzhou, CN-341000 China; 3 Deyang Company of Sichuan Provincial Tobacco Corporation, Deyang, CN-618400 China; 4 Hunan Tobacco Research Institute, Changsha, 410004 Hunan China; 5 Key Laboratory of Bio-resource and Eco-environment of Ministry of Education, College of Life Sciences, Sichuan University, Chengdu, CN-610065 China

**Keywords:** Cigar tobacco, Leaf morphological traits, QTL mapping, GWAS

## Abstract

**Background:**

Leaf morphology plays a crucial role in photosynthetic efficiency and yield potential in crops. Cigar tobacco plants, which are derived from common tobacco (*Nicotiana tabacum* L.), possess special leaf characteristics including thin and delicate leaves with few visible veins, making it a good system for studying the genetic basis of leaf morphological characters.

**Results:**

In this study, GWAS and QTL mapping were simultaneously performed using a natural population containing 185 accessions collected worldwide and an F_2_ population consisting of 240 individuals, respectively. A total of 26 QTLs related to leaf morphological traits were mapped in the F_2_ population at three different developmental stages, and some QTL intervals were repeatedly detected for different traits and at different developmental stages. Among the 206 significant SNPs identified in the natural population using GWAS, several associated with the leaf thickness phenotype were co-mapped via QTL mapping. By analyzing linkage disequilibrium and transcriptome data from different tissues combined with gene functional annotations, 7 candidate genes from the co-mapped region were identified as the potential causative genes associated with leaf thickness.

**Conclusions:**

These results presented a valuable cigar tobacco resource showing the genetic diversity regarding its leaf morphological traits at different developmental stages. It also provides valuable information for novel genes and molecular markers that will be useful for further functional verification and for molecular breeding of leaf morphological traits in crops in the future.

**Supplementary Information:**

The online version contains supplementary material available at 10.1186/s12870-024-05261-8.

## Introduction

Leaves are the main photosynthetic organs of plants. Leaf morphology is closely related to plant photosynthetic efficiency, transpiration, and gas exchange, which thereby affect plant biomass. Leaf development involves initiation of leaf primordium, establishment of leaf polarity, and leaf expansion, which is regulated by various factors including developmental signals and environmental conditions [1].

With the development of Next-Generation Sequencing (NGS) technology, studies on Quantitative Trait Locus (QTL) mapping has made great progress, and the QTLs for many important agronomic and economic traits of crops have been mapped. This makes it possible to study the genetic basis of complex quantitative traits, laying the foundation for map-based gene cloning. QTL mapping of some leaf size-related traits in crops have been studied. Jiang et al. (2022) identified five QTL regions regulating leaf size in Alfalfa (*Medicago sativa* L.) [2]; By combining RIL (Recombinant Inbred Line) and IF_2_ (Immortalized F_2_) populations, Liu et al. (2017) mapped QTLs for maize leaf width [3]; In addition, QTLs for flag leaf length, flag leaf width and flag leaf area in japonica rice were mapped by linkage analysis and Genome-Wide Association Studies (GWAS) using 2 RILs and a natural population [4]. Therefore, combining linkage analysis and GWAS to conduct QTL mining is an effective approach for unraveling the genetic basis of leaf morphological characteristics.

Tobacco is widely used as a model plant for fundamental biological research. *Nicotiana tabacum* L. (common tobacco) is important economic crop and is commonly classified into several categories based on the type and curing process, i.e. Flue-cured tobacco, Sun-cured tobacco, Burley tobacco, Cigar tobacco and Oriental tobacco. A cigar is made up of three parts: wrapper, binder and filler [5]. As the outermost part, wrapper tobacco leaves should be flat, wide and thin, with few visible veins; the optimal leaf vein angle is 45^°^~70^°^. Therefore, cigar tobacco is a good system to unravel the molecular mechanisms regulating leaf development due to its leaf morphological differences from other varieties. In previous studies, multiple QTLs associated with leaf traits of cigar tobacco have been identified on almost all chromosomes using QTL mapping analysis [6]. However, there have been no studies using GWAS, not to mention the combination of these two methods for revealing the genetic basis of leaf morphological traits in cigar tobacco.

In this study, germplasm collection of cigar tobacco containing 185 accessions and an F_2_ population consisting of 240 individuals derived from two cigar tobaccos were created and used for whole-genome resequencing. Association analysis and QTL mapping were performed using phenotyping data of leaf morphological characters, including leaf flatness and thickness, lateral vein diameter and leaf vein angle, obtained at different developmental stages for three consecutive years in two places. We subsequently combined the two mapping methods and selected the most significant SNPs for linkage disequilibrium (LD) analysis. By considering the leaf-specific expression and gene functional annotations, 7 candidate genes were predicted to be associated with leaf thickness. Our results provide a genetic basis for subsequent gene function verification and for molecular breeding of leaf morphological traits in cigar tobacco as well as other crops in the future.

## Materials and methods

### Plant materials and phenotype measurements

For the natural population, 340 cigar tobacco accessions collected worldwide were first screened phenotypically as well as using SSR markers. A total of 185 representatives (Supplementary Table 1) were selected and used in this study [7, 8]. All the 185 cigar tobacco germplasms have been deposited in the National Mid-term Genebank for Tobacco which is a collection of tobacco germplasm resource hosted at the Chinese Academy of Agriculture Sciences. The natural population was planted in Shandong (SD), China (120.45°E, 36.38°N) and Sichuan (SC), China (104.16°E, 31.13°N) in three consecutive years (2019–2021) with three biological replicates and 20 plants per replicate for each accession. Segedinska Ruca, which is characterized by flat and thick leaves, fine lateral veins and large leaf vein angles, was selected as the male parent P_1_ for crossing with the female parent Xiawanna which was designated as P_2_ and exhibited the opposite phenotype as P_1_, i.e., crinkly and thin leaves, thick lateral veins and small leaf vein angles. The F_2_ population was subsequently obtained and consisted of 240 individuals. Together with P_1_, P_2_ and F_1_ plants, F_2_ population were subsequently planted in SD, China, with three biological replicates and 50 individuals for each replicate.

The leaf morphological phenotypes of the natural population, leaf flatness, leaf thickness, lateral vein diameter and leaf vein angle were measured at flowering time. The parents, F_1_ and F_2_ populations were characterized for leaf traits at the rosette stage, budding time and flowering time. The phenotyping methods used were described previously [9]. Briefly speaking, the largest middle leaf was measured using surface roughness meter, angle ruler, vernier caliper and leaf thickness tester (Model No. YH-1) for leaf flatness, leaf vein angle, lateral vein diameter and leaf thickness, respectively.

### Whole-genome resequencing, SNP detection and annotation, and genotyping

Young leaves at the rosette stage were sampled and quickly frozen in liquid nitrogen. DNA was extracted by the SLS method and a sequencing library was constructed using an Illumina TruSeq Library Construction Kit. Whole-genome resequencing (WGS) was performed using fastq files following the default parameters, and the sequences were subsequently aligned to the reference genome of *N. tabacum* L. var ZY300 using BWA. SNP detection was performed using SAMTools (parameters: rmdup; [10]). ANNOVEAR [11] was used to annotate the detected SNPs.

### Genome-wide association analysis

The best linear unbiased prediction (BLUP) was calculated using the R package *lme4* (CRAN-Package lme4 (r-project.org)). A mixed linear model (MLM) was used to adjust the population structure [12]. Moreover, the Bonferroni correction method was used to derive a genome-wide significance threshold.

### Construction of the genetic linkage map

To construct a linkage map, we selected sites fixed for alternative alleles between the two parents. JoinMap 4.1 was used to rank the markers of each linkage group, and linkage maps were drawn using the Perl SVG module [13]. QTL mapping was performed using the composite interval mapping (CIM) method [14] implemented in WinQTLCart (version 2.5; [15]). The significance threshold was determined using 1000 permutation tests with MapQTL [16].

### KASP tag-based genotyping

The 100–200 bp up- and downstream sequences of the SNPs of interest were used for KASP primer design (Supplementary Table 2). KASP-PCR was performed on a 384-well PCR instrument (Eppendorf Mastercycler pro). The KASP-PCR products were read with a BMG POLARstar Omega scanner. The SNP typing results were analysed using KlusterCaller 3.4.1 software (Kincioscience). The aggregated genotypes near the X or Y axis were alleles linked to the FAM or HEX fluorescent tag sequences, respectively.

## Results

### Both the natural and F_2_ populations are suitable for standard genetic analysis

**Fig. 1:**
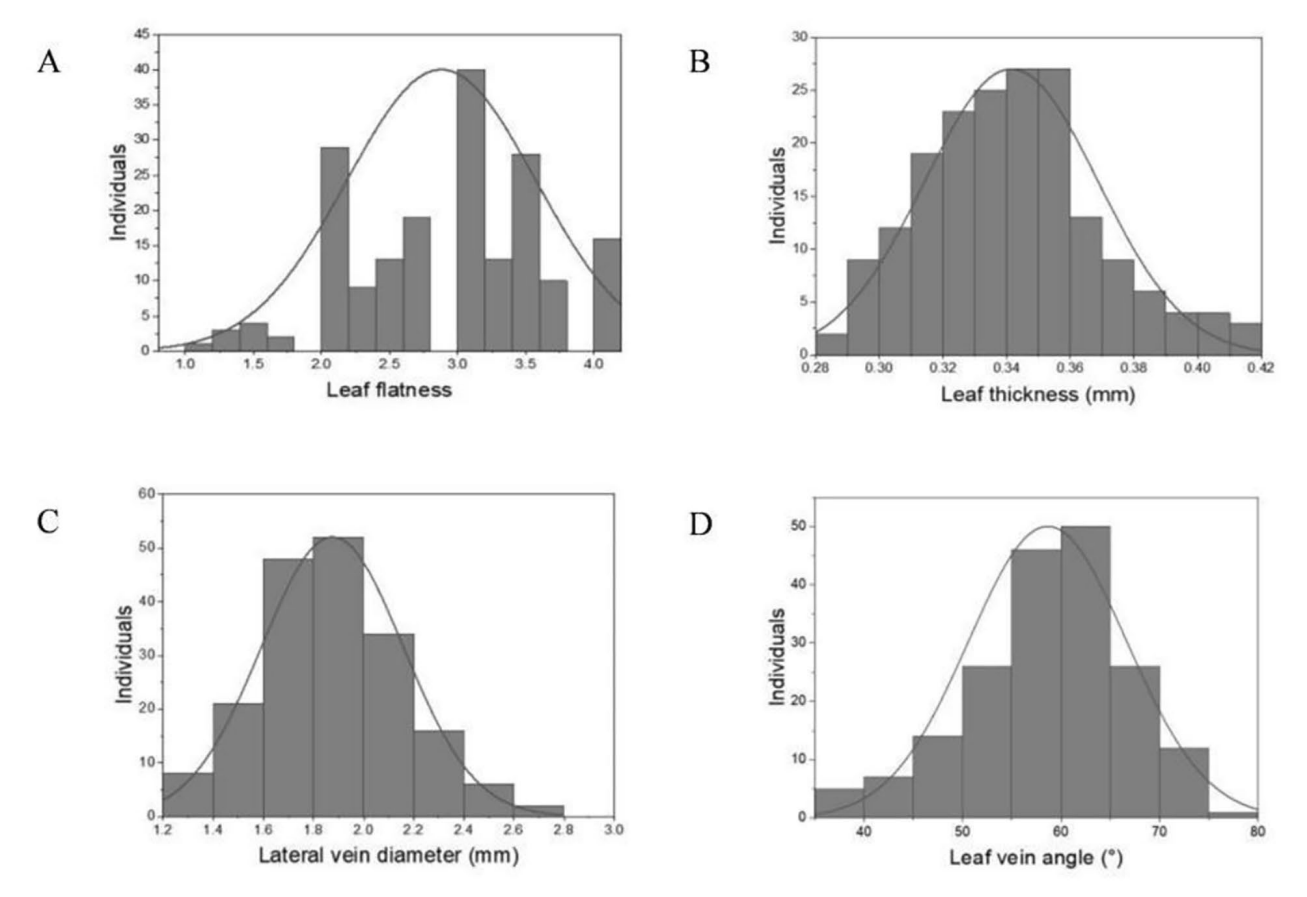
Distribution of phenotypic data regarding leaf flatness (**A**), leaf thickness (**B**), lateral vein diameter (**C**) leaf vein angle (**D**) in the natural population

In the natural population, the leaf morphological traits on average in three consecutive years at two different locations exhibited a normal distribution (Fig. 1), with a large degree of dispersion (Table 1). Therefore, the plant materials used as natural populations were suitable for subsequent genetic analysis.

**Table 1:** Phenotypic data analysis of leaf morphological traits in the natural population

Trait	Max	Min	Mean	SD	CV (%)	h^2^ (%)
Leaf flatness	4	1	2.89	0.69	23.88	94.46
Leaf thickness (mm)	0.83	0.28	0.35	0.08	22.86	84.37
Lateral vein diameter (mm)	2.4	1.24	1.77	0.185	10.45	29.87
Leaf vein angle (°)	77.29	39.58	61.34	6.3	10.27	90.42

**Fig. 2:**
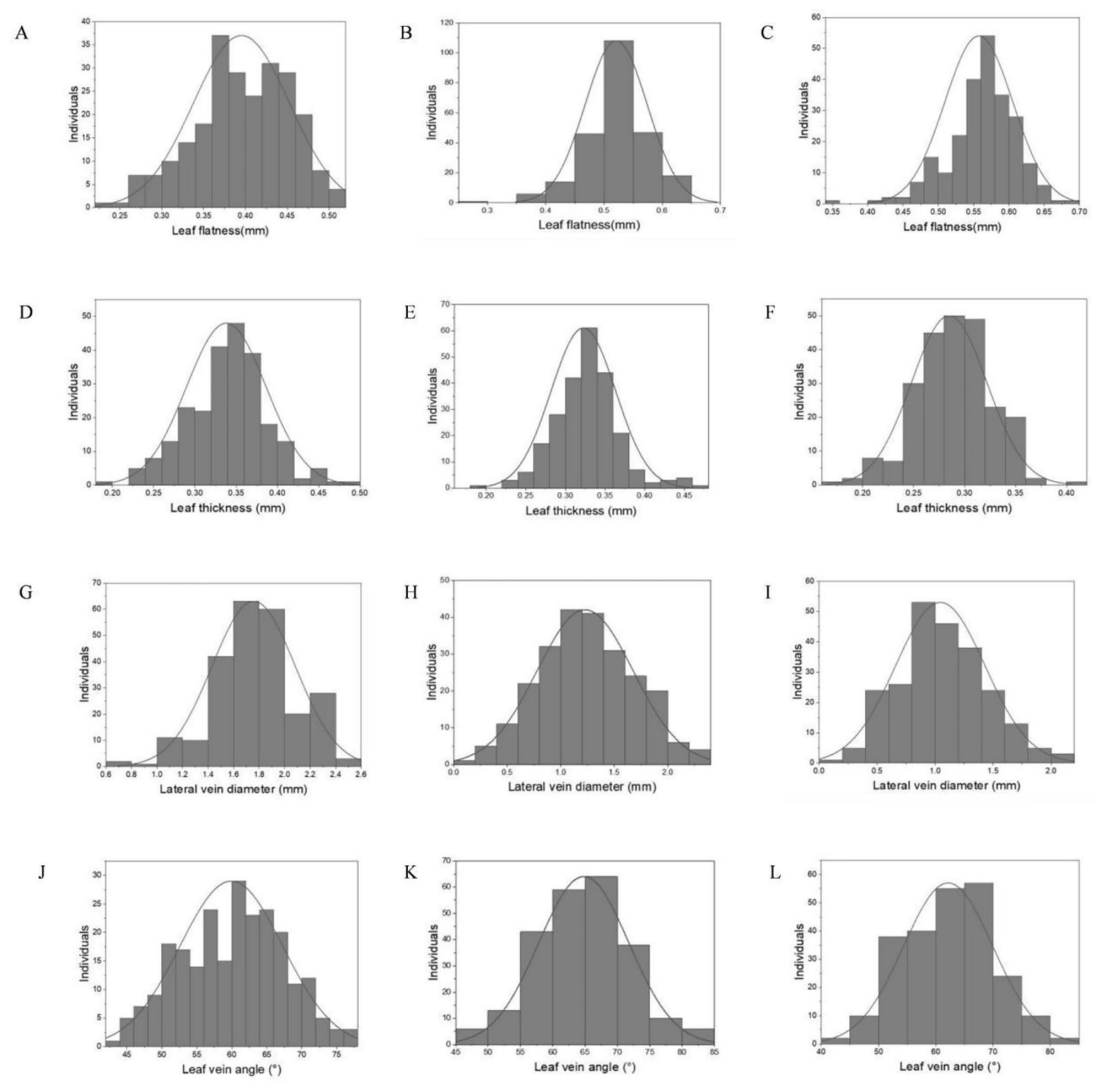
Distribution of phenotypic data in the F_2_ population. Leaf flatness (**A**, **B**, **C**), leaf thickness (**D**, **E**, **F**), lateral vein diameter (**G**, **H**, **I**) and leaf vein angle (**J**, **K**, **L**) were measured at the rosette stage, budding time and flowering time, respectively

The significance of leaf morphological traits between the two parents was also analysed (Table 2). Except for leaf thickness at the rosette stage, there were significant differences in all leaf traits between the two parents at the three developmental stages (*P* < 0.01), and all the leaf traits investigated showed a certain range of variation (8.61%~37.03%), indicating that the F_2_ population is also a qualified genetic population. Moreover, all the leaf traits exhibited a normal distribution at different developmental stages in the F_2_ population, which suggested that these traits are suitable quantitative traits and could be used for further genetic analysis (Fig. 2).

**Table 2:** Phenotypic data analysis of leaf traits in the F_2_ population

Trait	Period	P_1_	P_2_	F_1_	F_2_				
		Mean	Mean	Mean	Max	Min	Mean	SD	CV (%)
Leaf flatness (mm)	Rosette stage	0.46^B^	0.51^A^	0.52	0.51	0.24	0.40	0.06	14.57
	Budding time	0.48^C^	0.53^B^	0.55	0.65	0.28	0.52	0.05	10.23
	Flowering time	0.54^B^	0.57^A^	0.56	0.69	0.35	0.56	0.05	8.61
Leaf thickness (mm)	Rosette stage	0.40^A^	0.39^A^	0.36	0.48	0.19	0.34	0.05	14.29
	Budding time	0.28^B^	0.30^A^	0.30	0.47	0.18	0.32	0.04	12.73
	Flowering time	0.26^B^	0.30^A^	0.27	0.40	0.17	0.28	0.04	13.19
Lateral vein diameter (mm)	Rosette stage	1.78^B^	1.64^C^	1.97	2.53	0.70	1.76	0.33	18.58
	Budding time	1.47^A^	1.02^B^	1.13	2.33	0.13	1.23	0.45	36.25
	Flowering time	1.42^A^	1.06^B^	1.15	2.03	0.17	1.04	0.39	37.03
Leaf vein angle (°)	Rosette stage	57.18^C^	66.69^A^	61.48	77.51	43.30	59.93	7.36	12.29
	Budding time	54.48^B^	70.11^A^	67.78	82.24	47.03	64.81	6.95	10.73
	Flowering time	50.85^B^	68.71^A^	67.44	81.03	43.15	62.15	7.81	12.56

Note: The different capital letters represent significant differences between P_1_ and P_2_ (*P* < 0.01)

### Significant SNPs associated with leaf morphological traits were detected via GWAS

**Fig. 3:**
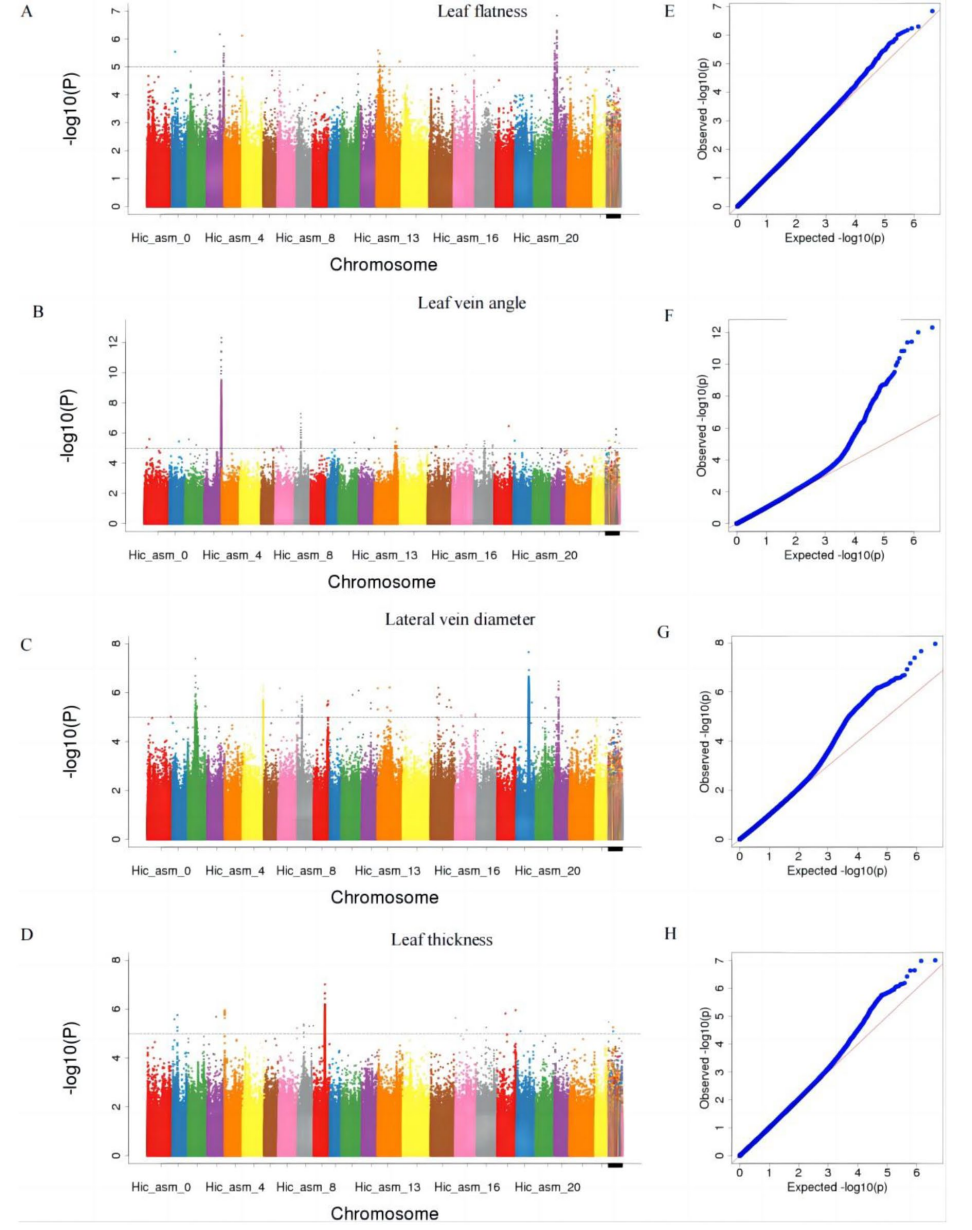
Manhattan plot (BLUP) and QQ plot for leaf flatness (**A**, **E**), leaf vein angle (**B**, **F**), lateral vein diameter (**C**, **G**) and leaf thickness (**D**, **H**)

A total of 2,119,142 high-quality SNPs were obtained by WGS of 185 accessions in the natural population and were found to be distributed almost evenly on 24 chromosomes, where one SNP appeared in every 440 bp. The numbers of SNPs of different types are shown in Supplementary Table 3. Manhattan plots (BLUP) and QQ plots for each phenotype generated through GWAS analysis are presented in Fig. 3. The significant SNPs shown in the Manhattan peaks are summarized in Supplementary Table 4. Next, we examined the location of these SNPs to determine whether any loci co-mapped in the QTL intervals were identified in the following QTL mapping analysis.

### Construction of a high-density genetic linkage map in cigar tobacco

**Fig. 4:**
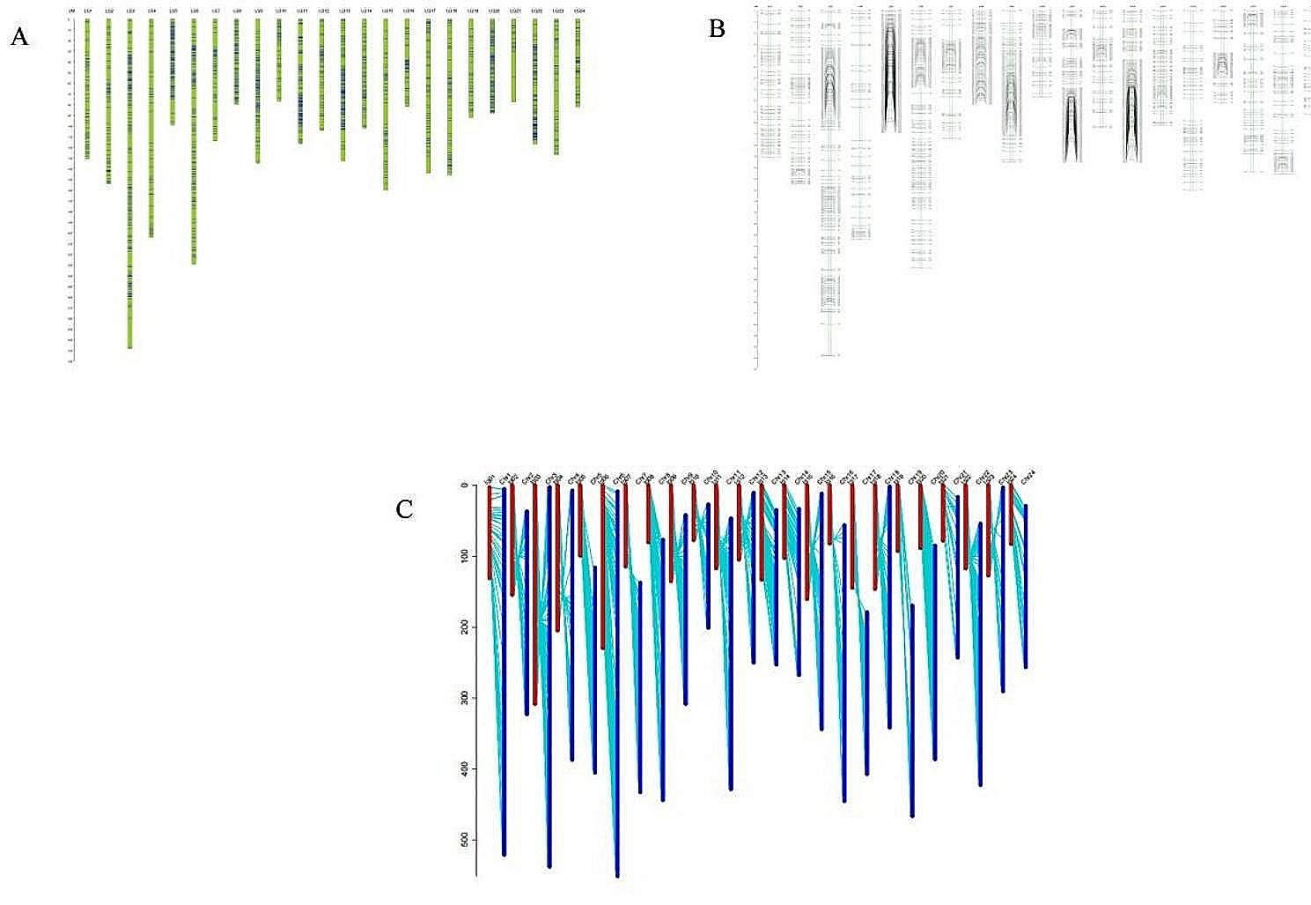
High-density genetic map of cigar tobacco **(A-B)** and collinearity analysis between the genetic and physical maps **(C)**. (**A-B**) The mapped SNP markers are designated as horizontal blue lines (**A**) and black lines (**B**). The X- and Y-axes show the linkage group ID and genetic distance, respectively. (**C**) The genetic map is designated as red lines, and the physical map is designated as blue

The whole genome of the F_2_ population was resequenced to construct a genetic linkage map of cigar tobacco. A total of 1,747,238 SNP markers were obtained, among which 311,687 were of the “aa × bb” type (Supplementary Table 5). After marker screening and filtering, 2,114 of the remaining ones were used for linkage analysis. In the end, the genetic map constructed contained 1682 SNP markers, with a total genetic distance (GD) of 3107.13 cM and an average inner-marker distance of 1.85 cM. Among the 24 linkage groups (lg), the maximum gap between markers was only 30.12 cM. Overall, the density of the genetic map was high (Table 3; Fig. 4A-B).

**Table 3:** The genetic map information

Group	SNP number	GD (cM)	Average distance (cM)	Max gap (cM)
lg01	53	130.78	2.47	10.93
lg02	58	154.23	2.66	15.15
lg03	171	308.33	1.8	27.7
lg04	42	204.54	4.87	30.12
lg05	109	99.21	0.91	13.62
lg06	121	229.54	1.9	10.43
lg07	48	114.25	2.38	14.98
lg08	80	80.08	1	4.52
lg09	97	135.11	1.39	11.28
lg10	40	77.05	1.93	6.4
lg11	107	116.8	1.09	11.78
lg12	42	104.47	2.49	12.42
lg13	118	132.84	1.13	11.1
lg14	57	102.42	1.8	7.61
lg15	50	160.18	3.2	20.17
lg16	41	81.97	2	12.99
lg17	55	144.26	2.62	16.35
lg18	61	146	2.39	11.82
lg19	30	92.46	3.08	10
lg20	105	88.33	0.84	2.82
lg21	17	77.79	4.58	23.95
lg22	108	117.05	1.08	9.16
lg23	36	127	3.53	18.89
lg24	36	82.44	2.29	11.91
total	1682	3107.13	1.85	30.12

We then performed collinearity analysis between the genetic map and physical map of ZY300, as shown in Fig. 4C. It was found that each linkage group had high collinearity with the corresponding chromosome, indicating the high quality of the genetic map constructed.

### QTL mapping of leaf morphological traits in the F_2_ population

A total of 24 QTL sites, for which LODs ranged from 2.81 to 4.85, were detected in the F_2_ population and were associated with the four leaf morphological traits during the three developmental periods studied (Table 4). Several QTL intervals were repeatedly detected for different traits and at different periods.

Among them, the interval from 41.9 to 46.3 cM on Hic_asm_0, associated with leaf thickness at flowering time, had the largest phenotypic contribution (80.25%), indicating its potential as a determining factor regulating the leaf thickness phenotype. One interval, located between 38.4 and 40.1 cM on Hic_asm_0 and associated with leaf thickness, was co-mapped at both the rosette and flowering times. The QTL 13.7 cM ~ 27.4 cM on Hic_asm_12, associated with the leaf vein angle phenotype, overlapped with the QTL 26.4 ~ 34.0 cM on the same chromosome and was linked with the leaf flatness phenotype, indicating that there may be a pleiotropic QTL.

**Table 4:** The significant QTL intervals detected in the F_2_ population

Trait	Period	Chr	Position(cM)	Interval (cM)	Physical Position	LOD	Add	PVE%
Leaf flatness	Rosette stage	Hic_asm_19	139.21	135.6 ~ 144.2	616,802~ 7,118,497	3.14	0.0144	8.30
Leaf flatness	Budding time	Hic_asm_22	118.01	117.1 ~ 123.8	94,565,950~ 208,405,639	2.81	0.025	8.13
Leaf flatness	Flowering time	Hic_asm_12	30.11	26.4 ~ 34	55,174,619 ~ 0.78649198	3.58	-0.0123	8.36
Leaf flatness	Flowering time	Hic_asm_12	40.01	35 ~ 42	28,057,339~ 46,162,324	2.87	-0.0111	7.08
Leaf flatness	Flowering time	Hic_asm_14	32.81	32.3 ~ 33.8	150,432,258 ~ 150,945,922	4.25	-0.0362	8.82
Leaf flatness	Flowering time	Hic_asm_14	39.01	37.2 ~ 41.4	138,110,740 ~ 147,501,716	3.73	-0.0366	5.67
Leaf flatness	Flowering time	Hic_asm_14	42.91	42.4 ~ 53.5	94,135,576~ 152,290,658	4.85	-0.039	6.55
Leaf flatness	Flowering time	Hic_asm_14	56.91	56.5 ~ 61.3	81,090,940~ 84,699,828	2.88	0.0312	7.61
Leaf thickness	Rosette stage	Hic_asm_0	38.91	38.4 ~ 40.1	177,071,232 ~ 178,462,185	2.52	-0.057	72.29
Leaf thickness	Flowering time	Hic_asm_0	39.81	36.1 ~ 41.6	164,734,782 ~ 182,927,010	3.97	-0.0078	26.05
Leaf thickness	Flowering time	Hic_asm_0	43.81	41.9 ~ 46.3	173,142,563 ~ 202,812,801	3.23	0.0063	80.25
Leaf thickness	Flowering time	Hic_asm_0	49.11	47.8 ~ 50	198,822,069 ~ 199,942,926	3.98	0.0066	69.92
Leaf thickness	Flowering time	Hic_asm_0	50.81	50 ~ 52.9	195,509,942 ~ 200,643,451	3.83	0.0036	25.89
Leaf thickness	Flowering time	Hic_asm_0	58.61	54.7 ~ 61.6	188,605,498 ~ 192,482,610	3.28	0.0023	13.61
Leaf thickness	Flowering time	Hic_asm_0	62.71	62 ~ 66.9	189,427,758 ~ 189,587,929	3.7	-0.0051	21.97
Leaf thickness	Flowering time	Hic_asm_21	44.21	43 ~ 45.5	67,864,283~ 68,601,626	2.67	-0.0357	21.75
Leaf thickness	Flowering time	Hic_asm_21	66.11	64 ~ 67	61,501,500~ 81,162,967	3.19	0.0218	5.85
Leaf thickness	Flowering time	Hic_asm_21	92.21	92.1 ~ 93.7	43,636,432~ 44,902,552	2.54	-0.0268	9.64
Leaf thickness	Flowering time	Hic_asm_21	103.31	99.1 ~ 105.4	36,178,893~ 42,590,969	4.75	-0.0268	19.02
Lateral vein diameter	Budding time	Hic_asm_3	43.91	39.8 ~ 48.2	96,592,730~ 124,071,750	3.89	0.1812	10.86
Lateral vein diameter	Budding time	Hic_asm_3	54.91	53.1 ~ 56.9	29,365,320~ 58,455,819	4.16	0.1898	13.15
Lateral vein diameter	Flowering time	Hic_asm_8	0.01	0 ~ 11	133,979,690 ~ 137,457,470	2.61	0.1381	9.19
Leaf vein angle	Rosette stage	Hic_asm_12	22.31	13.7 ~ 27.4	75,742,165~ 91,061,317	2.69	0.7363	5.29
Leaf vein angle	Flowering time	Hic_asm_23	79.71	73.4 ~ 80.7	97,090,840~ 102,504,794	3.26	-1.162	7.23

### Co-mapping combining the GWAS and QTL mapping results

The physical positions of the significant SNPs and flanking markers of the QTLs were compared using the ZY300 genome. Most importantly, several significant SNPs were found to be located within the QTL intervals (Table 5). Twelve SNPs associated with leaf thickness co-localized within the QTL between MK163 and MK211. The consistency of the GWAS and QTL mapping results indicated the importance of co-mapped SNPs as potential genetic loci regulating corresponding phenotypes.

**Table 5:** The significant SNPs located within the QTL intervals

Trait	Method	chr.	Marker	Position	Marker	Position
Leaf thickness	QTL mapping	Hic_asm_0	Mk163	173,142,563	Mk211	202,812,801
Leaf thickness	GWAS	Hic_asm_0		186,470,318		
		Hic_asm_0		187,486,936		
		Hic_asm_0		188,121,291		
		Hic_asm_0		188,299,181		
		Hic_asm_0		188,354,210		
		Hic_asm_0		188,424,753		
		Hic_asm_0		188,634,387		
		Hic_asm_0		188,854,129		
		Hic_asm_0		189,866,579		
		Hic_asm_0		191,463,977		
		Hic_asm_0		191,811,074		
		Hic_asm_0		192,639,125		

### Predicted candidate genes associated with leaf thickness in cigar tobacco

**Fig. 5:**
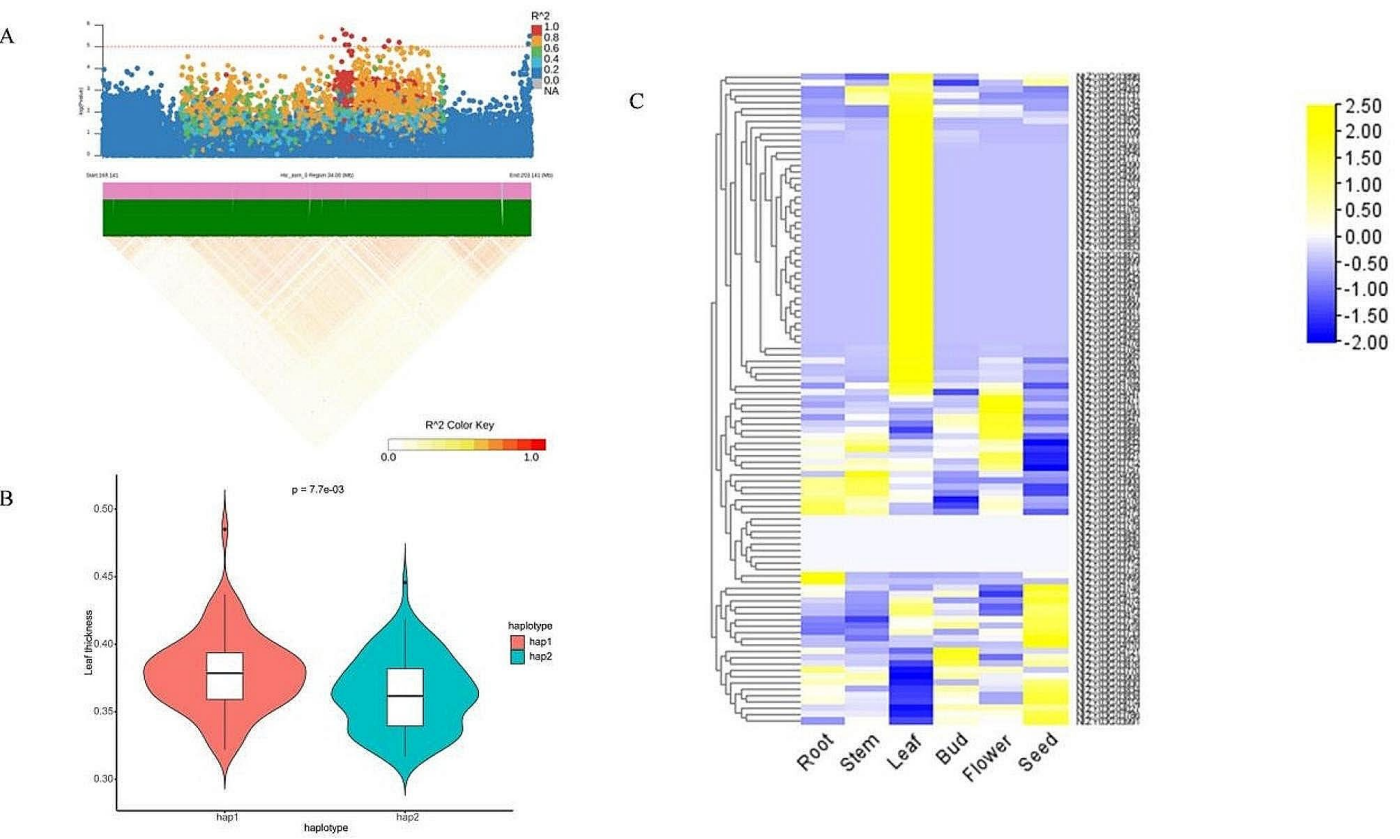
LD plot (**A**) and halpotype analysis (**B**) of the co-mapped QTL region, and heatmap of leaf thickness-related gene expression in various tobacco tissues (**C**). (**A**) Genomic region of chromosome Hic_asm_0:178.144-198.139 Mb for leaf thickness is shown in a Manhattan plot and LD heatmap. **(B)** Haplotype analysis of the co-mapped QTL related to leaf thickness is shown in a violin plot. **(C)** Expression data were obtained from a publicly available database. Rows represent 103 co-mapped genes that contained missense variance

To further narrow down the selected QTL region and screen potential candidate genes, a LD plot of the co-mapped region of chromosome Hic_asm_0 was created. A region containing 428 genes with a size of 20 Mb (Hic_asm_0: 178.144-198.139 Mb) was found to be linked (Fig. 5A). To validate the regulatory role of the co-mapped region regarding the leaf thickness phenotype, a haplotype analysis was further performed. The significant difference of leaf thickness between two haplotypes was shown in Fig. 5B. Among the genes detected, 103 genes exhibited *missense* variance, of which 47 genes were expressed in leaves 2 times higher than in other tissues (Fig. 5C).

Leaf thickness is often associated with cell size, cell number, relative vacuole volume, and cell wall composition [17–19]. At the molecular level, the process of leaf development involves coordinated regulation among small RNAs, transcription factors and hormones. Based on the functional annotations and relevant literature, 7 genes were predicted to be candidate genes related to the leaf thickness phenotype (Table 6) and were considered very informative for exploring the molecular mechanism controlling leaf thickness in the future.

**Table 6:** Predicted candidate genes associated with leaf thickness in cigar tobacco

Gene ID	Gene name	GO/KEGG description	Classification of SNP	Location
*NtZY03G03765*	unknown	TORC1 complex	missense	Hic_asm_0:173,455,249 ~ 173,457,457
*NtZY03G03853*	unknown	Mitotic-specific cyclin-2-like	missense	Hic_asm_0:180,951,865 ~ 180,952,630
*NtZY03G04001*	unknown	Mitotic-specific cyclin-2-like	missense	Hic_asm_0:193,787,888 ~ 193,788,916
*NtZY03G03722*	Far1-related sequence 10	Far1-related sequence 7-like isoform X1	missense	Hic_asm_0:171,055,252 ~ 171,056,787
*NtZY03G03943*	unknown	Pectinesterase inhibitor 18-like	missense	Hic_asm_0:187,824,447 ~ 187,824,791
*NtZY03G04003*	Extensin-3-like	Inward rectifier potassium channel activity	missense	Hic_asm_0:193,825,501 ~ 193,840,877
*NtZY03G03751*	unknown	Cytochrome P450 71A1-like	missense	Hic_asm_0:172,769,599 ~ 172,769,937

### KASP tag developed for leaf trait genotyping in cigar tobacco

**Fig. 6:**
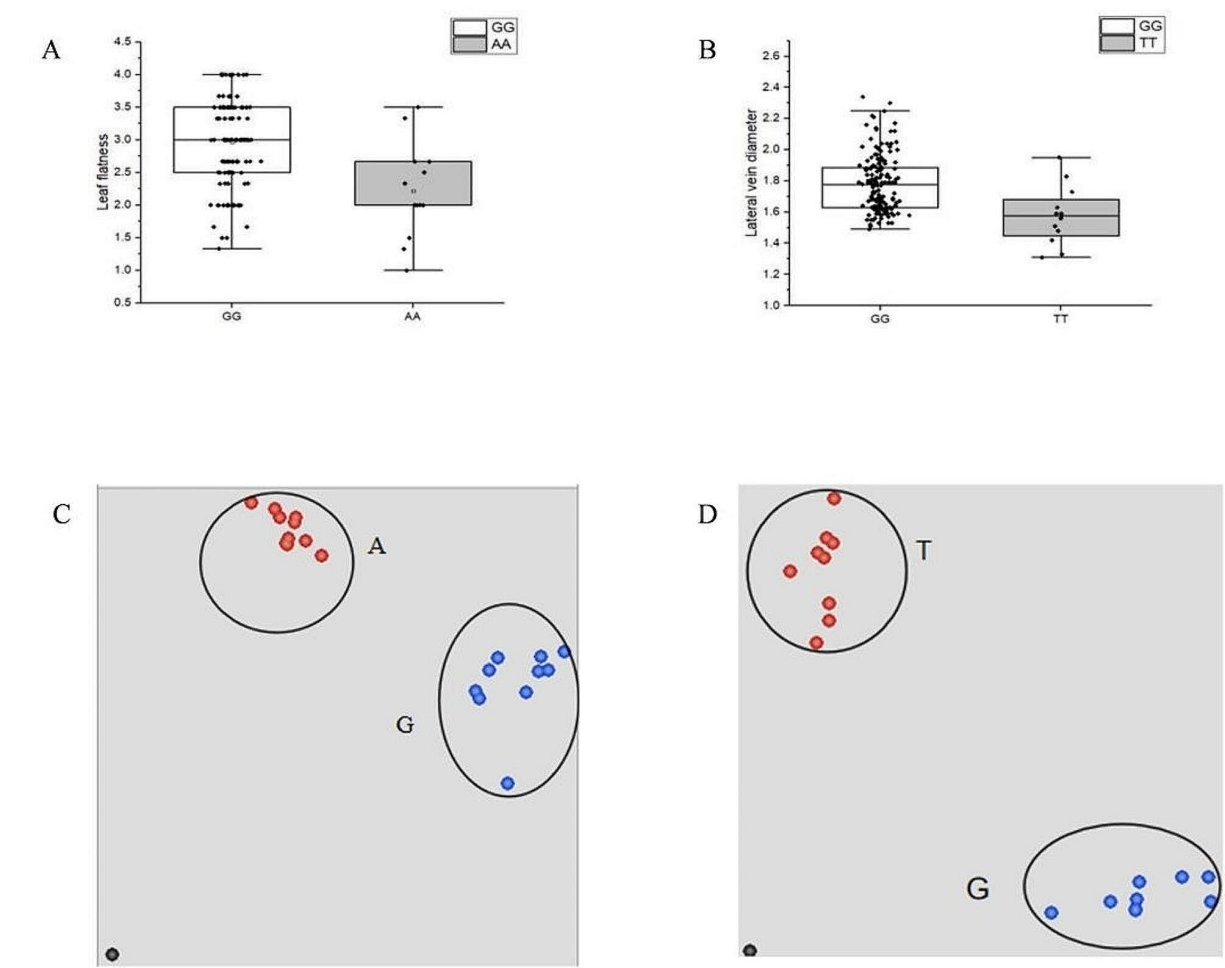
The distribution of phenotypic data associated with the markers LF11 (**A**) and LVD07 (**B**) and the genotyping results for LF11 (**C**) and LVD07 (**D**). The genotypes aggregated near the X or Y axis were alleles linked to FAM or HEX fluorescent tag sequences, respectively

To test whether some SNPs could be applied for genotyping leaf morphological traits in unknown cigar tobacco materials, two significant SNPs detected via GWAS were selected for designing Kompetitive Allele Specific PCR (KASP) markers. The germplasms separated by the genotypes showed significant difference in the phenotypic data (Fig. 6A-B). In the end, the markers LF11 and LVD07 were developed for leaf flatness and lateral vein diameter, respectively (Supplementary Table 6). To verify the KASP markers, a number of germplasms (Supplementary Table 7) were used for genotyping. The results showed that both the LF11 and LVD07 primers could distinguish the two homozygous genotypes, with one blue signal converged near the X-axis and the other red signal near the Y-axis (Fig. 6C-D). Therefore, both tags were developed successfully and could be used for leaf morphological trait genotyping in cigar tobacco plants in the future.

## Discussion

Cigars, as rolled-leaf tobacco products, require cigar wrappers with flat and thin leaves combined with fine lateral veins and large leaf vein angles. The phenotypic differences between wrapper tobaccos and other varieties make it a good model plant for studying the genetic basis of leaf mophological characters. In this study, we used both GWAS and QTL mapping analyses and co-mapped one QTL region to predict causal genes regulating the leaf thickness trait.

Compared with those of other crops, gene mapping studies of tobacco started relatively late, mostly focusing on disease resistance and yield [20, 21]. Our study is the first to identify the loci and candidate genes associated with the leaf morphological trait in cigar tobaccos. In this study, we preliminarily screened the QTLs responsible for leaf morphological traits through both linkage mapping and GWAS analysis in cigar tobacco. For the leaf thickness phenotype, one QTL within a 30.8 Mb interval (Hic_asm_0: 175.087-205.851 Mb) from QTL mapping was found co-mapped with the one of 20 Mb (Hic_asm_0: 178.144-198.139 Mb) identified by GWAS. Applying of both analysis successfully fine-mapped the QTL intervals, meanwhile increased mapping accuracy. Besides the co-mapped region, some QTLs or SNPs were only detected in one analysis, which suggests either it is a rare allele or no segregation happened at this region in F_2_ population.

Leaf thickness is an important morphological characteristic not only in cigar wrapper breeding but also strongly correlated with plant strategy in response to environmental variables such as high light and CO_2_ concentrations [22, 23], cold [24–26] and drought [27]. However, the molecular mechanism regulating leaf thickness is poorly understood. In dicots, leaf thickness is related to mesophyll cell size, cell number and the number of cell layers [28]. Thicker leaves tend to have more chloroplasts per cell and more stroma and thylakoid membranes, all of which determine leaf photosynthetic capacity [29].

TARGET OF RAPAMYCIN (TOR), which encodes a highly conserved Ser/Thr kinase, has been reported to regulate leaf cell size as a positive regulator in Arabidopsis [30, 31]. TOR might directly control cell cycle regulators in leaf development, and surprisingly, cyclin B (CYCB), which partially constitutes the plant cell cycle machinery, was also detected from the co-mapped QTL. The *cycb* mutant exhibited a reduced leaf area, possibly due to decelerated cell cycle progression [32]. In addition to direct influences on cell size and cell number, there are several regulators involved in chloroplast division that ultimately affect cell expansion and cell division. Disruption of *FAR1-RELATED SEQUENCE4* (*FRS4*), a transposase-derived transcription factor, results in enlarged chloroplasts through transcriptional regulation of *ACCUMULATION AND REPLICATION OF CHLOROPLASTS5* (*ARC5*), which is essential for chloroplast division [33, 34]. Furthermore, leaf thickness has been reported to be positively related to cell wall thickness [35]. Therefore, several genes related to the metabolism of cell wall components, such as *pectinesterase inhibitor 18-like, Extensin-3-like* and *Cytochrome P450 71A1-like* involved in the lignin biosynthetic pathway, were also selected for further functional verification.

## Conclusions

In summary, the leaf morphological traits of cigar tobacco, especially leaf thickness, were studied via two combined forward genetic methods, GWAS and QTL mapping. Nine significant SNP peaks and 24 QTL sites associated with four leaf morphological traits were detected in the accessions and F_2_ population, respectively. A comparison of the two sets of results revealed a co-localized leaf thickness-related genomic region containing 103 genes which had the missense changes. Through gene expression analysis and interpretation of the gene functional annotation, 7 genes were predicted to be potential functional genes controlling the leaf thickness phenotype during leaf development in tobacco plants. In addition, two KASP markers linked with leaf flatness and lateral vein diameter were developed and verified in some cigar germplasms. These results will lead to the identification of novel genes and molecular markers that will be valuable for further functional verification and for molecular breeding of leaf morphological traits in crops in the future.

### Electronic supplementary material

Below is the link to the electronic supplementary material.

Supplementary Material 1

## Data Availability

The data that support the findings of this study are included in the manuscript. The all sequence data was uploaded to NCBI with accession code PRJNA1119079.

## Abbreviations

GWAS: Genome-wide association analysis
QTL: Quantitative trait locus
LD: Linkage disequilibrium
NGS: Next-generation sequencing
RIL: Recombinant Inbred Line
IF_2_: Immortalized F_2_
WGS: Whole-genome resequencing
BLUP: Best linear unbiased prediction
MLM: Mixed linear model
CIM: Composite interval mapping
GD: Genetic distance
lg: Linkage groups
KASP: Kompetitive Allele Specific PCR
TOR: Target of rapamycin
CYCB: Cyclin B
FRS4: FAR1-related sequence4
ARC5: Accumulation and replication of chloroplasts
